# Actionable Items to Address Challenges Incorporating Peer Support Specialists Within an Integrated Mental Health and Substance Use Disorder System: Co-Designed Qualitative Study

**DOI:** 10.2196/17053

**Published:** 2020-11-26

**Authors:** Margaret Almeida, Annie Day, Bret Smith, Cynthia Bianco, Karen Fortuna

**Affiliations:** 1 The Mental Health Center of Greater Manchester, ProHealth NH Manchester, NH United States; 2 Harvard University Cambridge, MA United States; 3 The Mental Health Center of Greater Manchester, Peer Collaborative Manchester, NH United States; 4 Department of Psychiatry Dartmouth College Concord, NH United States

**Keywords:** experience-based co-design, mental health recovery, substance-related disorders, peer support, peer support specialist, health workforce, patient experience, patient satisfaction, coproduction

## Abstract

**Background:**

Peer support specialists offering mental health and substance use support services have been shown to reduce stigma, hospitalizations, and health care costs. However, as peer support specialists are part of a fast-growing mental health and substance use workforce in innovative integrated care settings, they encounter various challenges in their new roles and tasks.

**Objective:**

The purpose of this study was to explore peer support specialists’ experiences regarding employment challenges in integrated mental health and substance use workplace settings in New Hampshire, USA.

**Methods:**

Using experience-based co-design, nonpeer academic researchers co-designed this study with peer support specialists. We conducted a series of focus groups with peer support specialists (N=15) from 3 different integrated mental health and substance use agencies. Audio recordings were transcribed. Data analysis included content analysis and thematic analysis.

**Results:**

We identified 90 final codes relating to 6 themes: (1) work role and boundaries, (2) hiring, (3) work-life balance, (4) work support, (5) challenges, and (6) identified training needs.

**Conclusions:**

The shared values of experience-based co-design and peer support specialists eased facilitation between peer support specialists and nonpeer academic researchers, and indicated that this methodology is feasible for nonpeer academic researchers and peer support specialists alike. Participants expressed challenges with agency restrictions, achieving work-life balance, stigma, and low compensation. We present actionable items to address these challenges in integrated mental health and substance use systems to potentially offset workforce dissatisfaction and high turnover rates.

## Introduction

### Background

Peer support specialists have a vital role in delivering new models of integrated mental health and substance use care. Peer support specialists are individuals with lived experience of a mental health condition or substance use disorder, or both, who are trained to provide support services (or “peer support”) to others with similar challenges [1-3]. Peer support services augment traditional psychiatric care and have been shown to be effective in reducing stigma, psychiatric distress, and hospitalizations among service users [4-6]. Knowing the value of peer support services, 46 states across the United States have implemented Medicaid-reimbursable peer support specialist training programs and services to develop a peer support specialist workforce of approximately 30,000 individuals [1-3]. Despite the benefits of peer support specialists to the mental health and substance use disorder system, peer support specialists in the United States have reported job dissatisfaction, which has resulted in high turnover rates [6,7].

Of note, one of the major challenges identified in the scientific literature is a general lack of understanding among peer support specialists of their role within the mental health system, resulting in feelings of exclusion [8,9]. The US National Association of Peer Supporters has developed supervision guidelines to offset these challenges in mental health systems [10] Yet, as peer support specialists are now increasingly being incorporated into *integrated* mental health and substance use systems, it is not known if additional challenges have surfaced.

### Objective

As peer support specialists are considered essential workers during the COVID-19 pandemic and are part of a fast-growing mental health and substance use workforce in integrated care settings, it is imperative to understand challenges in their role in order to develop systems to support peer support specialist workforce satisfaction and retention. The purpose of this study was to explore peer support specialists’ experiences regarding employment challenges in various integrated mental health and substance use workplace settings.

## Methods

### Experience-Based Co-Design Methodology

Using the experience-based co-design (EBCD) methodology, peer support specialists and academic researchers collaborated as equal partners. EBCD is a participatory action research method used for collaboratively improving health care services with academic researchers and service users working as partners in improvement in health care [11,12]. EBCD has been shown to be an effective method of services improvement in health care, as it facilitates the process to identify and address health care workforce culture, values, and behaviors [11,13].

The Manchester Peer Collaborative partnered with nonpeer academic researchers using EBCD and discussed peer support specialists’ challenges in the integrated mental health and substance use workplace. The Peer Collaborative meets in person monthly and comprises 15 peer support specialists from New Hampshire, USA. Each of the 15 peer support specialists provides varying services, including traditional mental health peer support and integrated mental health and substance use peer support services. Conversations and concerns were brought forward by peer support specialists who were aware of potentially detrimental work experiences within the peer support specialist local work landscape. The Peer Collaborative had previously discussed major areas of concern, including high turnover rates, challenges with various staffing issues, and receiving supervision from traditionally trained clinical staff versus supervision from another peer support specialist. The Peer Collaborative wanted to further understand the issues and work experiences of locally employed, peer support specialists in order to improve the overall work environment. Bringing this to the attention of leadership, there was agreement that further resources and examination were needed. These initial conversations between nonpeer academic scientists and the Peer Collaborative and agency leadership led to this project’s co-designed main objective: to explore peer support specialists’ experiences regarding employment challenges in various integrated mental health and substance use workplace settings in New Hampshire.

### Data Collection

Using a convenience sample, 2 trained peer support specialist research partners contacted 5 agencies employing peer support specialists via telephone. They described the purpose of the study to agency peer supervisors and assessed the agency’s interest in having peer support specialists participate in a 90-minute focus group on-site. Next, the peer support specialist research partners scheduled focus groups. To reduce the burden, focus groups were scheduled for convenient times at the agency where focus group participants worked.

Peer support specialist research partners conducted 3 focus groups with 5 peer support specialists in each focus group (N=15), each lasting approximately 90 minutes. The focus groups were audio recorded. Peer support specialist research partners co-designed the focus group interview guide. Participants received no compensation to participate. Peer support specialist research partners were paid their normal rates. We conducted focus groups until no new information or themes were brought forward by the focus group (ie, until saturation was met; data saturation happens during qualitative research in which no new information is discovered from interviews or focus groups—this indicates to researchers they can stop collecting data) [14]. To reduce bias and allow for participants to speak openly, we held focus groups in private rooms without management personnel present. Before the start of each focus group, a verbal scripted consent form was handed out to participants and read aloud by peer support specialist research partners.

### Focus Group Interview Guide Development

Peer support specialist research partners collaboratively developed questions for the focus groups with the principal investigator (PI; MA). Questions were based on peer support specialist research partners’ experience as peer support specialists offering services within a variety of integrated mental health and substance use systems. The question topics asked about interviewing, hiring, training, and conducting peer support services across various integrated mental health and substance use systems. By developing the questions and an interview guide with peer support specialist research partners, we increased the likelihood of promoting objectivity and including culturally informed questions [15].

### Peer Support Specialist Research Partner Training

Peer support specialist research partners independently completed an institutional review board (IRB) training online. To further support their research partner role, the PI trained peer support specialist research partners to conduct research activities to ensure the development of necessary interview skills (eg, drawing out reliable information from the focus group participants) [16]. The training was conducted over 6 weekly meetings in approximately 90-minute blocks of time each. Specifically, peer support specialist research partners were trained in the following areas: research ethics and conflicts of interest, recruitment methodology, qualitative interview guide development, qualitative data collection, qualitative data analysis, manuscript writing, and dissemination. All training was offered in person, and information was presented to peer support specialist research partners verbally as well as with written material to support reinforcement of in-person training. Peer support specialist research partners practiced conducting and leading mock focus groups [17,18]. We conducted 2 mock focus groups in which peer support specialist research partners alternated being the mock note taker or facilitator role. The PI observed and provided feedback to support learning.

### Ethical Considerations for Co-Designing With Peer Support Specialist Research Partners

We submitted a summary of the study protocol to all participating agencies and met with leaders as follow up for questions and feedback. At the lead agency, this study was first reviewed by the clinical services team, consisting of clinical directors from various service departments (eg, adults, children, emergency services, acute services). Nonpeer directors who supervise and employ peer support specialists were part of the review. Of note, we asked peer support specialist employees from multiple agencies to provide their input regarding setting-specific workforce challenges and to offer solutions to those challenges. As such, it was important to ensure that each agency agreed to provide a safe, confidential, open communication environment, to which all of the agencies agreed. In addition, we requested from each agency that supervisors of the participating peer support specialists not be present during focus groups and that a private room be available for the focus group to take place in. Furthermore, we requested a waiver of written consent. We submitted a scripted consent form to the IRB that researchers could hand out and read aloud prior to the beginning of each focus group obtaining verbal consent versus written consent to participate. The co-design team further protected the identity of participants in case of disagreement or repercussions by not collecting any individual identifiers (eg, name, email) in data collection.

After the review and discussion, a vote was taken and the study was approved with a letter of support generated and addressed to the PI. We then submitted this study for external ethics board review after the participating agencies submitted letters of support for the project. The review was conducted and approvals obtained by the New Hampshire IRB, which was the IRB of record for all agencies involved.

### Data Analysis

After each focus group ended, 2 peer support specialist research partners and the PI met in person for 30 minutes to debrief and discuss the focus group themes and exchange ideas. Together, they discussed the most important themes and how each agency presented similar or different information [14].

Next, we transcribed audio recordings. The analyses of focus group data was informed by conducting a content analysis and then thematic analysis [19]. We reviewed and followed focus group analysis guidelines to ensure consistency in the transcript review process [20]. An initial meeting was conducted to review the entire transcript as a group. The PI and peer support specialist research partners then separately read the transcripts while taking notes and documenting impressions. We categorized relevant topics, themes, patterns, or other topics that were unexpected or recognized as important by participants. The peer support specialist research partners and the PI held 2 group data analysis meetings to review categories and discuss key themes emerging from the data and to achieve final consensus.

## Results

### Participants and Themes

All 5 agencies we contacted responded positively and invited every peer support specialist at the agency to attend 1 of the 3 focus groups; however, 2 agencies were unable to participate due to scheduling conflicts and staffing shortages, resulting in a 60% agency response rate.

The focus groups consisted of participants (N=15) employed as a peer support specialist by 1 of the 3 agencies: (1) a peer-led agency with a primary focus on substance use disorder recovery (n=5, 33%), (2) a peer-led agency focused on mental health wellness and recovery (n=5, 33%), or (3) an agency-led community mental health center (n=5, 33%). All agencies also offered various integrated mental health and substance use support and services. Of the participants, 53% (n=8) were male and 87% (n=13) were White; their ages ranged from 21 to 60 years. The majority of participants had received a 2- or 4-year college degree (n=8, 53%), 3 participants had received their high school diploma (20%), 2 participants had received some education after high school (13%), and 1 participant had received a master’s degree (7%).

We identified 90 final codes relating to 6 themes: (1) work role and boundaries, (2) hiring, (3) work-life balance, (4) work support, (5) challenges, and (6) identified training needs.

### Peer Work Role and Boundaries

The first theme was related to peer work roles and boundaries. Participants noted that they were not trained specifically to maintain clear boundaries with service users. Participants reported that they were trained to operate with flexibility based on the need of the service user. For example, following peer training, if a service user was feeling lonely, that could mean a peer support specialist could socialize with the service user (eg, going fishing together, or go out to eat); however, this would be considered unethical if a clinician were to conduct these activities. This boundary issue is highlighted in one participant’s statement:

You don’t want to live with regret about drawing a line in the sand and hold your boundaries and something catastrophic happens. It’s a really slippery slope because it really sometimes is life or death of a client.

Participants indicated they would be reprimanded if they conducted activities that violated clinical professional boundaries—not peer professional boundaries.

Participants also commented that they worked closely with clinicians who were often not familiar with peer specialist work activities or their role, and that the clinicians expected activities that were not in the job description. For example, participants reported they had been asked to feed a service user’s cat or do an emergency room clinical assessment. Participants reported they were often misunderstood to be junior clinicians or community health workers. Agency-led employed participants added that it is important that all clinical supervisors take training on how to work with peers and that agencies need to train providers on understanding the peer role.

### Hiring Peer Support Specialists

The second theme was related to hiring. Participants agreed that before hiring a peer support specialist, it is important to know that the potential peer employee is far enough along in their own recovery and are also able to have enough personal resources to give support to somebody else, and the ability to feel comfortable working with people who are in recovery. Peer support specialists agreed that recovery time alone is not the most important measure of recovery, that “Quality is more important than quantity.” Participants employed at the peer-operated agencies were more comfortable hiring individuals who were still struggling with a substance use disorder and who may be at risk for reoccurrence. They felt that offering a “get-well” position is an incentive to help people get and stay in recovery. Get-well jobs can be paid or unpaid positions that are usually a less-demanding role just after achieving sobriety. These positions have manageable hours and fewer responsibilities to facilitate a pattern of showing up to work [21].

An emerging finding from the participants suggested the importance of knowing what is expected in the job and that discussing the idea of becoming a professional peer with a trusted friend, other peer specialist, a therapist, or recovery coach for outside perspective could be helpful. Agency-run organizations were more likely to have more requirements upon hiring. The agency-operated peer specialists reported that their agency made a point to hire those already certified or individuals working toward certification for Certified Recovery Support Worker and Certified Peer Support; however, participants noted it is not uncommon for other agencies to ask for existing recovery time prior to hiring.

Important hiring interviewing questions that we identified included asking potential hires what they are doing for their own recovery. Participants reported they would feel comfortable if a potential employer asked them about their own recovery. However, they stated they would feel uncomfortable if a nonpeer interviewer asked them about their personal recovery. One male participant explained that a new peer at a hiring interview can be “intimidated talking to nonpeers about their low times or legal issues to someone other than a peer.”

The majority of participants reported that peers should be involved in the hiring process. In fact, 1 participant was already part of the hiring process. Specifically, participants suggested their role in hiring should include developing interview questions, selecting interviewees, and asking and interpreting interview questions. Peer support specialist involved in hiring would look for an employee with passion, altruism, self-reflection, and finding their own happiness through service to others. Peer support specialists would also look for hiring red flags such as not being able to identify one’s potential reoccurrence triggers, not being mindful of their own interpersonal boundaries, not having dedicated time for self-care activities, and expecting significant financial gain.

Participants reported the need for supervisory support once hired. Participants reported that supervisors can be a peer themselves or a clinical staff member. Supervisors’ characteristics should include honesty and respect, celebration of a peer support specialist’s strengths, flexibility, and willingness to refine job skills after mistakes are made. Supervisors should also offer trainings.

### Challenges Peer Support Specialists Experience in Clinical Environments

The third theme was related to challenges peer support specialists experience in the clinical environment. This theme comprised 3 subthemes: stigma, work-life balance, and low salary coupled with high job demands.

#### Stigma

All participants agreed that stigma associated with having a mental health condition or substance use disorder is the number 1 challenge they face as a peer support specialist. As peer support specialists, they, themselves, experience stigma, as well as observing it directed toward those they assist. Participants reported that stigma and the fear of the unknown are top reasons people do not seek help. One female participant explained:

Peers are not taken seriously, despite solid outcomes, and we are people with lived experience, so we carry that stigma of mental illness.

Another male participant explained:

There is internal stigma and external stigma and the health care community itself sometimes contributes to ongoing stigma.

Participants suggested the need for widespread education toward changing the culture of people who are not affected by a mental health condition or substance use disorder.

#### Work-Life Balance

Participants emphasized the importance of work-life balance. This subtheme was related to maintaining a work-life balance and avoiding burnout. Participants employed at a peer-led agency focusing primarily on substance use disorder recovery reported challenges with work-life balance. One participant reported:

We all work from home. We all take calls on our days off. We all do it.

They acknowledged that their agency’s work that focused on the community’s opioid crisis had left staff experiencing vicarious trauma. This compelled peer staff to be available when they, themselves, were not scheduled to work and were at home.

To address vicarious trauma, participants reported that peers checked in with one another, were vigilant if a peer colleague appeared exhausted and worn out, and sent tired peer colleagues home when rest was needed. They reported having access to supervisors regularly and participating in debriefings as needed. Participants reported they relied on and trusted one another to speak up and notice when a colleague was experiencing workplace burnout. All participants reported the importance of agency flexibility to send a peer home for self-care when needed.

#### Low Salary Coupled With High Job Demands

Low salary was a reported challenge. Participants reported lower hourly salary than for other positions even though there is still an ongoing shortage of peer support specialists at the agency. This shortage was especially prominent on the intensive treatment teams. Participants noted that 1 peer support specialist as assigned to each team, leaving them to cover large numbers of individuals. Participants suggested that a cost-effectiveness study may help show the value of peer services and impact salaries.

### Training Needs of Peer Support Specialists

Self-care and other trainings were identified training needs. All participants identified training on self-care as the top training need. Additional needed trainings identified were how to cope with vicarious trauma, receiving updates on the topic of sex trafficking in their community, how to work with the chronic homeless population, what resources and strategies are available to peer support specialists working with perpetrators, and providing trauma-informed care. Participants wanted trainings on how to work with clinicians and their roles. They felt it was important as part of the agencywide orientation that all clinical positions have a training on this. One participant stated that peers “need training on how to work with clinicians and clinicians need training on how to work with a peer.”

## Discussion

### Principal Findings

The aim of this study was to investigate peer support specialists’ experiences regarding training, recruiting, hiring, management, work roles, and retention in the integrated mental health and substance use disorder workplace by partnering with peer support specialist experts using the EBCD methodology. Peer support specialists identified challenges with agency restrictions, achieving work-life balance, stigma, and low compensation. Peer support specialists detailed previously unidentified actionable items to address workforce challenges, including hiring procedures and trainings.

Peer support specialists in integrated agencies experienced challenges similar to those faced by peer support specialists in nonintegrated settings. Participants reported challenges with boundaries, work-life balance, and experiencing personal stigma in the workplace and confirmed the ongoing challenge of low compensation. This finding is consistent with the role of peer support specialists in nonintegrated agencies [22-24]. Participants reported stigma as the biggest challenge, either by experiencing stigma themselves, sometimes even from their own professional colleagues, or observing it directed toward those they assist. Challenges associated with stigma have been documented in previous studies [24], and a variety of strategies exist to create safe zones for people experiencing mental illness in the peer support agencies [25]. The peer support profession is not well understood across various agencies. Supervisory and leadership training for both clinicians and peers may educate agencies on the role of peer support specialists and also prepare peers to work with clinicians. Peer support specialists and nonpeer support specialists employed in integrated settings may not be receiving adequate training conducive to their roles. Improving and offering consistent training standards statewide could potentially improve the work experiences of peer support specialists, particularly in integrated clinical settings.

Training in self-care is paramount to being a caregiver, human services worker, or health care worker. Peer support specialists encounter this same need. Self-care training, while offered frequently, not only needs to be formally tailored to peer support specialists, but also should include a structure of support built into peer training programs. Peers in previous studies had reported challenges with work-life balance and burnout leading to negative outcomes [26]. To address and prevent the common experience of burnout and vicarious trauma that peer support specialists encounter while working alongside first responders and crisis workers, participants recommended a supportive organizational structure and specialized training in these areas.

Agencies looking to hire peer support specialists should involve already employed peer support specialists in the hiring process. Inclusive hiring policies and practices have been documented as an identified organizational characteristic indicating readiness to hire peer workers [27]. Participants provided guidance on what to look for in a new peer support specialist to find the right fit for the position and to identify interviewees who were (1) able to identify and hence avoid or manage potential reoccurrence triggers, (2) mindful of their own interpersonal boundaries, (3) capable of dedicating time for self-care activities, and (4) not expecting significant financial gain but to be rewarded by the role of helping others.

The shared values of EBCD and peer support specialists eased facilitation between peer support specialists and nonpeer academic researchers toward a mutual goal. EBCD and peer support specialist practice standards share a similar values system, including that (1) people from all backgrounds can provide knowledge, (2) people can give practical help to each other that provides mutual benefit to both parties, (3) individuals seeking services are equal to the care provider or academic researcher, and (4) experiential knowledge is valued [13,28]. These shared values are similar to that of a peer support values system that also is based on experiential knowledge, inclusion of all people, and mutuality [3]. Peer support specialist research partners expressed satisfaction with regard to the EBCD process and finding answers to the questions that they had regarding the peer support specialist’s work experience.

### Limitations and Strengths

We acknowledge that this study had several limitations. First, this study included a small convenience sample; however, we collected data until saturation was met. Second, this study was racially homogeneous, which may not be a good representation of a racially and ethnically diverse population response. Data saturation may have been met due to the racially and ethnically homogeneous population. As such, these findings should be interpreted with caution in generalizing to a racially and ethnically diverse population. Third, respondents may have felt pressure to give similar answers to the moderator’s questions depending on the group dynamics in the focus groups. The peer support specialist research partners may have presented a bias by encouraging or discouraging answers with body language or voice inflection. Peer project leads were trained to mitigate bias by discussing common examples of interviewer bias to increase their awareness as part of their focus group training with the PI, and they were unpaid volunteers. The PI listened to the audio recording and did not identify any verbal biases. As peer support specialists are increasingly involved as research partners by taking on researcher roles, scientifically exploring methods to mitigate peer support specialist interviewer bias may help advance the role of peers as equal partners in research. Fourth, peer support specialist research partners wanted participants to remain completely autonomous; as such, this study did not collect participant names and demographics beyond what was reported, or match participants using a study ID.

While this study had limitations, a strength of this study was the use of EBCD approach to engage with peer support specialists and identify previously unidentified methods to address workforce challenges related to hiring procedures and trainings. This report can be used to guide the advancement of the peer workforce. The project’s subject matter was also focused on peer support specialists in the integrated mental health and substance use disorder peer support services field, which is an area that requires continuing attention from the academic and health care communities.

### Dissemination to Stakeholders

Upon completion of this study, the peer support specialist research partners (AD, BS) and the PI submitted a report of the focus group results to agency leadership. In addition, we set up a meeting with stakeholders, state peer support specialist leadership, and local agency leadership, allowing for further discussion and sharing of the focus group results that was conducted by the peer project lead support specialist (AD, BS). An ongoing effort with state and local leadership has been to bring awareness to the current national versus local practice. The Peer Collaborative met with state leadership and submitted a multiday training outline for peer support specialists in a community mental health setting. The training included an integrated care focused section. In these meetings we discussed that oversight at the state level for peer support specialists would be better served by a separate peer board than under an existing state-licensed alcohol and drug counselor board for peer support specialist expertise and workforce standardization. We will continue to use focus group findings to facilitate solutions identified by peers within the integrated mental health and substance use disorder systems.

### Conclusions

This study produced actionable insights affecting the mental health and substance use disorder system from the perspective of peer support specialists. Participants expressed challenges with agency restrictions, achieving work-life balance, stigma, and low compensation. Participants’ recommendations related to training, hiring procedures, management, work roles, and retention in the mental health and substance use disorder workplace may offset these challenges and work toward advancing the peer workforce. The shared values of EBCD and peer support specialists eased facilitation between peer support specialists and nonpeer academic researchers and indicate that this methodology is feasible for nonpeer academic researchers and peer support specialists alike. The partnership established collaboration and equality among research team members allowing for multiple areas of expertise to enhance research in the peer support specialist workforce field.

## Abbreviations

EBCD: experience-based co-design
IRB: institutional review board
PI: principal investigator
